# Using Smartphone Sensors for Ataxia Trials: Consensus Guidance by the Ataxia Global Initiative Working Group on Digital-Motor Biomarkers

**DOI:** 10.1007/s12311-023-01608-3

**Published:** 2023-11-28

**Authors:** Andrea H. Németh, Chrystalina A. Antoniades, Juergen Dukart, Martina Minnerop, Clara Rentz, Bart-Jan Schuman, Bart van de Warrenburg, Ilse Willemse, Enrico Bertini, Anoopum S. Gupta, Carlos Bandeira de Mello Monteiro, Hajar Almoajil, Lori Quinn, Susan B. Perlman, Fay Horak, Winfried Ilg, Andreas Traschütz, Adam P. Vogel, Helen Dawes, on behalf of the AGI Digital-Motor Biomarkers Working Group

**Affiliations:** 1 Nuffield Department of Clinical Neurosciences, University of Oxford, Oxford, UK; 2 Oxford Centre for Genomic Medicine, Oxford University Hospitals NHS Foundation Trust, Oxford, UK; 3 Neurometrology Group, Nuffield Department of Clinical Neurosciences, University of Oxford, Oxford, UK; 4 Institute of Neuroscience and Medicine, Brain & Behaviour (INM-7), Research Centre Jülich, Jülich, Germany; 5 Institute of Systems Neuroscience, Medical Faculty & University Hospital Düsseldorf, Heinrich Heine University Düsseldorf, Düsseldorf, Germany; 6 Institute of Clinical Neuroscience and Medical Psychology, Medical Faculty & University Hospital Düsseldorf, Heinrich Heine University Düsseldorf, Düsseldorf, Germany; 7 Department of Neurology, Center for Movement Disorders and Neuromodulation, Medical Faculty & University Hospital Düsseldorf, Heinrich Heine University Düsseldorf, Düsseldorf, Germany; 8 Institute of Neuroscience and Medicine, (INM-1), Research Centre Jülich, Jülich, Germany; 9 Friedreich Ataxie Förderverein E.V, Pliening, Germany; 10 Department of Neurology, Donders Institute for Brain, Cognition, and Behaviour, Radboud University Medical Center, 6525 Nijmegen, Netherlands; 11 Donders Institute for Brain, Cognition and Behaviour, Radboud University Medical Center, Nijmegen, Netherlands; 12 Unit of Neuromuscular and Neurodegenerative Disorders, Dept Neurosciences, Bambino Gesu’ Children’s Research Hospital, IRCCS, Rome, Italy; 13 Department of Neurology, Massachusetts General Hospital and Harvard Medical School, Boston, MA USA; 14 Faculty of Medicine, University of São Paulo, São Paulo, SP Brazil; 15 School of Arts, Science and Humanities, University of São Paulo, São Paulo, SP Brazil; 16 Physical Therapy Department, College of Applied Medical Sciences, Imam Abdulrahman Bin Faisal University, Damman, Saudi Arabia; 17 Department of Biobehavioral Sciences, Teachers College, Columbia University, New York, NY USA; 18 University of California Los Angeles, Los Angeles, CA USA; 19 Department of Neurology, Oregon Health & Science University, Portland, OR USA; 20 APDM Precision Motion, Clario, Portland, OR USA; 21 Section Computational Sensomotorics, Hertie Institute for Clinical Brain Research, Tübingen, Germany; 22 Centre for Integrative Neuroscience (CIN), Tübingen, Germany; 23 Research Division “Translational Genomics of Neurodegenerative Diseases”, Hertie Institute for Clinical Brain Research and Center of Neurology, University of Tübingen, Tübingen, Germany; 24 German Center for Neurodegenerative Diseases (DZNE), University of Tübingen, Tübingen, Germany; 25 Centre for Neuroscience of Speech, The University of Melbourne, Melbourne, Australia; 26 Division of Translational Genomics of Neurodegenerative Diseases, Hertie Institute for Clinical Brain Research, University of Tübingen, Tübingen, Germany; 27 Center for Neurology, University Hospital Tübingen, Tübingen, Germany; 28 Redenlab Inc, Melbourne, Australia; 29 NIHR Exeter Biomedical Research Centre, Medical School, Faculty of Health and Life Sciences, College of Medicine and Health, St Lukes Campus, University of Exeter, Heavitree Road, Exeter, UK

**Keywords:** Ataxia, Internal smartphone Sensors, Digital motor performance outcome measures

## Abstract

Smartphone sensors are used increasingly in the assessment of ataxias. To date, there is no specific consensus guidance regarding a priority set of smartphone sensor measurements, or standard assessment criteria that are appropriate for clinical trials. As part of the Ataxia Global Initiative Digital-Motor Biomarkers Working Group (AGI WG4), aimed at evaluating key ataxia clinical domains (gait/posture, upper limb, speech and oculomotor assessments), we provide consensus guidance for use of internal smartphone sensors to assess key domains. Guidance was developed by means of a literature review and a two stage Delphi study conducted by an Expert panel, which surveyed members of AGI WG4, representing clinical, research, industry and patient-led experts, and consensus meetings by the Expert panel to agree on standard criteria and map current literature to these criteria. Seven publications were identified that investigated ataxias using internal smartphone sensors. The Delphi 1 survey ascertained current practice, and systems in use or under development. Wide variations in smartphones sensor use for assessing ataxia were identified. The Delphi 2 survey identified seven measures that were strongly endorsed as priorities in assessing 3/4 domains, namely gait/posture, upper limb, and speech performance. The Expert panel recommended 15 standard criteria to be fulfilled in studies. Evaluation of current literature revealed that none of the studies met all criteria, with most being early-phase validation studies. Our guidance highlights the importance of consensus, identifies priority measures and standard criteria, and will encourage further research into the use of internal smartphone sensors to measure ataxia digital-motor biomarkers.

## Introduction 

Hereditary ataxias are relatively rare disorders with heterogenous clinical presentation and progression, as well as underlying genetic aetiology [1, 2]. With disease-modifying therapies on the horizon, there is a need for scalable, objective, reliable, sensitive, and specific outcome measures for upcoming trials to capture early disease progression and response to therapies during feasible short follow-up periods [3–5]. As sufficiently large patient cohorts may only be achieved in multi-centre trials including patients from diverse settings and geography, protocol harmonisation and agreement on clinical outcome assessments, including digital outcome measures, are an important aspect to consider for any such trials.

Motor abnormalities, particularly balance, coordination, speech, and eye movements are key features of all ataxias but are very variable due to the immense heterogeneity of ataxia subtype. For example, ataxia may cerebellar, sensory/afferent, or both, and there may be the presence or absence of confounding additional motor features, such as spasticity and dystonia. In addition, there is variability within different genetic subtypes, within different mutation classes, and even marked intrafamilial variability for the same mutation. Given the heterogeneity of ataxias, clinical trials are targeted to gene-specific subtypes. However, ultimately, personalised and detailed outcome measures will be required to capture these differences, requirements for which smartphones can offer solutions.

Motor abnormalities in ataxia have been evaluated extensively using laboratory-based systems. Optical motion capture systems, force plates, and saccadometers have all been recommended as gold standard measures for eye movements, gait, and standing balance, respectively [6, 7]. However, movement characteristics that are only measured in the laboratory are impractical for multisite clinical trials, limit participation, and frequency of measurement, and often do not reflect movement impairments in everyday life [8–10]. Clinical outcome assessments should truthfully and comprehensively measure the specified construct and demonstrate that it is discriminative, sensitive, reliable, and deemed feasible in terms of cost and time constraints for the purpose it is intended. However, it has also been highlighted by patients, their families, and clinical teams and endorsed by the FDA and other regulatory bodies, that it is critical to measure what is really bothering patients and when measuring performance outcomes in particular, that some of these should be of functional relevance [11]. There are a few sensors available that measure such functional activities, for example an instrumented feeding spoon, but these may be difficult to implement at scale [12].

The Ataxia Global Initiative (AGI) aims to provide consensus guidance to clinicians, academics, and industry that will enable International collaboration and align with regulatory recommendations, thereby progressing ataxias towards clinical trial readiness [1]. AGI has several Working Groups (WG) including WG4, which focuses on Digital-Motor Biomarkers, specifically focussing on four pre-defined key clinical domains, namely gait/posture, upper limb, speech, and oculomotor function. Our Smartphone Working Group works alongside these key clinical domains.

Rapid changes in digital measurement capabilities make the use of smartphone sensors an attractive, inclusive, and scalable possibility for measuring these domains in clinical trials, facilitating applicability across countries and different data collection environments.

All sensors, when used in clinical trials, need to confirm to robust standards including those set out by COSMIN and national/international guidelines (see URLs), such as privacy, security, accuracy, absolute and relative reliability, sensitivity, and clinical application. The use of smartphone sensors requires additional consideration of interoperability of data and how to handle variation that may be present from phone to phone. However, as long as each phone sensor behaves reliably, methods can be implemented that target within-person changes for use within trials. Their use for clinical trials has already been validated in other conditions [13].

Using smartphone sensors is particularly attractive as smartphones are in wide public use and the measures can be acquired regularly in laboratory/clinic and real-life community settings, providing more inclusive participation, ecologically valid data, and more frequent phenotyping to measure disease progression [8]. However, there is a need to determine core measures and develop protocols for their valid use within clinical trials. The AGI smartphone sub working group of WG4 was formed to highlight the benefits and challenges of using smartphone sensors, alongside other measurement strategies, as well as to integrate the critical work of the WG4 key domain subgroups.

Smartphones contain internal sensors including accelerometers, gyroscopes, global positioning (GPS) technology, as well as cameras, digital video capabilities, and microphones that may be used for both passive (continuous data acquisition in the background of daily life) and/or active (prescribed tasks requiring direct input from the user) data collections [14]. These sensors may also be situated within bespoke devices, with a number under validation for use in ataxias. These devices often offer more accurate solutions but tend to employ a greater number of sensors to provide accuracy at the expense of both usability and cost.

Smartphones can also be used to upload and manipulate data from external sensors. Although several studies have successfully used a range of sensors to measure and monitor characteristics of ataxia [12, 15–18], the use of internal smartphone sensors is an emerging measurement specialty, and few studies have specifically focused on these internal smartphone sensors, using either Android or iOS devices.

Furthermore, and importantly for future studies, there is currently no clear guidance on agreed measures or core criteria standards for using smartphone sensors as performance outcome measures in ataxia clinical trials and cohort studies.

This paper is the first to address this need and provides guidance for measures using internal smartphone sensors to assess performance outcome measures. These include standardised task(s) actively undertaken by a patient such as walking, limb movements, and speech. However, we acknowledge that the versatility of smartphones, which can operate many different apps, means that other clinical outcome measures including passive monitoring of movement, as well as patient-reported, observer-reported, and clinician-reported outcome measures could augment ataxia motor studies, although we do not make recommendations on those in this paper.

To generate guidance for the use of internal smartphone sensors as performance outcome measures for each of the recommended key domains we had the following objectives:

1. To identify current evidence for the use of internal smartphone sensors in different clinical ataxia domains (gait, upper limb, speech, and oculomotor), using a literature review.
2. To determine current smartphone use, identify any applications in development and gather opinion from interested stakeholders on proposals for future measures using smartphones through a survey of the AGI WG4 stakeholders (“Delphi 1”).
3. To determine consensus priorities for performance outcome measures obtained using internal smartphone sensors, through a survey of the AGI Digital-Motor Biomarker WG key domain leads (“Delphi 2”).
4. To agree on a set of standard criteria when using smartphone sensors for digital motor measures of ataxia which are provided herein as guidance, through Expert panel consensus with stakeholders, including PPI, clinicians, academics, and industry partners.
5. To determine how many current measures from publications identified in Stage 1 met the standard criteria recommended in objective 4, highlighting the utility for such criteria in the development of future research studies and trial design.

## Material and Methods

### Consensus Building

**Fig. 1:**
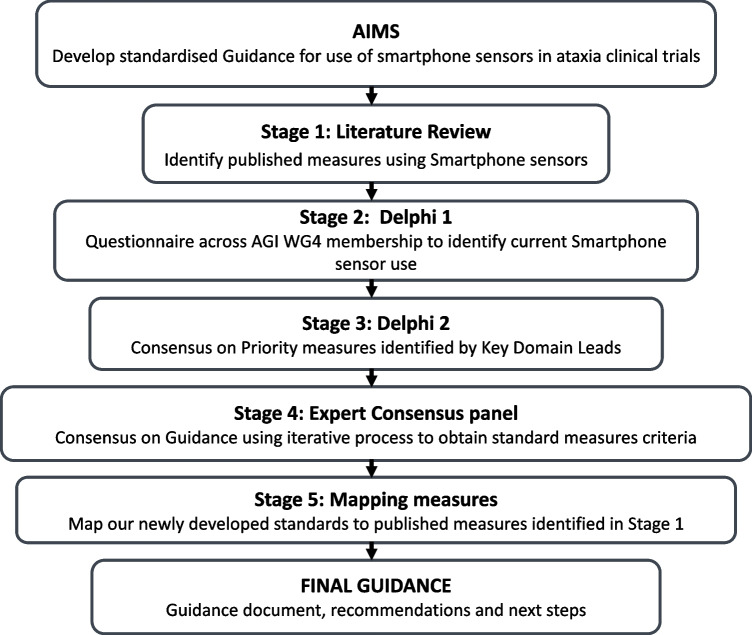
Visual representation of the five-stage process to develop Guidance for Smartphone Sensor Use in Ataxia Clinical Trials

Standardised methodologies were adapted to develop the guidance, including literature review, Delphi methodology, and monthly panel meetings in a 5-stage process to achieve consensus on standard criteria and priorities for ataxia smartphone assessments (see Fig. 1).

The initial step was the creation of a smartphone sub Working Group following discussion within the AGI Digital-Motor Biomarkers Working Group 4 (AGI WG4) and included experts representing the four pre-defined key domains who had an interest and expertise in smartphone sensor measurement or digital technologies for health. Further membership was encouraged across geographical regions to include members from Europe, North America, South America, Australasia, and across disciplines. Discipline experts included medically qualified clinicians (neurology, genetics), allied health professionals, academics with an interest and expertise in movement disorders including ataxias, patient representatives with lived experience of ataxias and industry partners. The key domain leads attended the online meetings to coordinate opinions from across AGI WG4. HD, AN, and CA created the Delphi 1 questionnaire. IW, BVDW, and CR designed and carried out the initial literature review to May 2021 and members of the Expert panel (HD, AHN, CAA, JD, IW, BVDW, MM, BJS) carried out an updated review to March 2023 in each of the respective domain areas. The Expert panel guided each stage of the process. The wider AGI group were consulted regularly through the AGI WG4 key domain leads. Expert external review was also sought from HA, CM, and LQ.

## Stage 1: Literature Review Data Sources and Searches

An extensive literature review was performed to identify publications relevant to internal smartphone sensors as defined above (3D spatial measures, camera/digital videos, microphones). The initial systematic search was based on a wider, parallel initiative by a subgroup of authors (IW, CR, BVDW) who identified publications covering smartphone or Tablet apps to monitor movement disorders until May 19th, 2022, contained within PubMed, Web of Science, Embase, and Cochrane. Key search words included “mobile applications”, “tablet”, and “smartphone” and were combined with keywords covering all primary movement disorders such as “movement disorders”, “ataxia”, and “tremor” and similar clinical descriptors. From the results of this search here, we include only publications on internal smartphone sensors relevant to ataxia. An expanded and updated search was then conducted by Expert panel members from May 2022 to April 2023 and included publication references, Apple’s AppStore and Google Play (App Store).

## Stage 2: Current Smartphone Use Across AGI

In Delphi 1, we surveyed the 80 person membership of the Digital-Motor Biomarkers Working Group of AGI. This stage was undertaken in order to identify a snapshot of interested Stakeholders and their demographics; current use of smartphone apps and sensors and their operating systems; current digital outcome measures in use and for which key domains or other measures; how many new smartphone Apps were in development; suggested Domain areas for future use; the potential patient reach of the WG for future clinical trials. We used an online Qualtrics questionnaire, Qualtrics™ survey software (Qualtrics XM Platform™, UT, USA) with closed questions such as “What are you already measuring with Smartphone technology?” and “What do you wish to measure using Smartphone technology” with fixed options for responses including gait/posture, upper limb, oculomotor, speech, physical activity, fitness metrics, and health metrics. In addition, we offered a freetext section for “other”. These questions were aimed at capturing measures that primarily aligned with the AGI WG4 key domains. In addition, we included another section enabling freetext responses to “Can you suggest which aspects of the condition you think should be measured/monitored regularly” which enabled us to capture other domains of interest, although we did not expand on this further, as this was beyond the scope of the current study. The data was captured within the Qualtrics software and then converted to graphical format within Microsoft Excel.

## Stage 3: Delphi 2 Consensus on Priority Measures from Key Clinical Domain Leads

Stage 1 (Literature review) and Stage 2 (Delphi survey to the AGI WG4 membership) revealed wide variability in both the methodologies using smartphones reported in the literature and a lack of consensus within AGI WG4 membership on exactly what should be measured and monitored, using internal smartphone sensors. Therefore, in Stage 3, (“Delphi 2”), domain leads from AGI WG4 were invited to provide one or two critical measurement priorities which should be included in ataxia smartphone assessments at the current time. The domain leads (for gait/posture, upper limb, speech, and oculomotor) developed their priorities based on the results from their own Working Group Data.

## Stage 4: Expert Consensus Panel to Agree Core Standard Criteria for Smartphone Measures

Monthly meetings of the expert consensus panel were carried out as an iterative process to develop and agree on each stage methodology and to gain consensus on key stage progressions. The team agreed on the term standard criteria for use in stage 5 and for the final guidance. Working definitions for these criteria were generated based on standard definitions, to facilitate their use and agree meaning by all stakeholders. The panel agreed on the final guidance.

The authors and key clinical domain leads including PPIE formed the Expert panel. Standard criteria were determined in a series of meetings of the Expert panel and are based on those criteria the group considered to be essential. They were based on international standards such as COSMIN and COMET principles and digital regulatory guidelines (see URLs).

## Stage 5: Mapping Smartphone Sensor Measures Reported in Published Data (Identified by Stage 1 Literature Review) onto Standard Criteria Developed in Stage 4

The publications identified in Stage 1 were evaluated to determine how the measures used in each research study mapped onto the standard criteria developed here. Final mapping was confirmed by the Expert panel.

## Results

### Stage 1: Literature Review

Seven publications were identified that explicitly investigated ataxia using internal smartphone sensors as defined above (i.e. accelerometer/gyroscope, in-built camera/digital video, microphone). These publications focus on the key clinical domains including three on gait/posture [15, 16, 19], one on upper limb [20], one on several SARA components (gait, upper limb, speech [21]), and two on oculomotor measures [22, 23].

Three publications reported newly devised smartphone apps. The upper limb assessment was based on an app called 15 White dots App-Coo-Test (WDACT) [20]. During the touchscreen test, the participant is asked to touch a white dot (appearing consecutively and randomly on the screen) as quickly as possible. For each upper limb (dominant and non-dominant hand), an average time, the standard deviation, and the coefficient of variation of the executed touchscreen trials are calculated as a measure of fine motor skills.

A variation of the WDACT, the App-Coo-Balance Test uses smartphone 3D accelerometers fixed to the lower trunk to characterise body sway while standing, as a measure of balance control. During the balance task, the smartphone sensors assess the oscillation of the trunk in both static positions (feet together, on a broad base, sitting) and dynamic balance (gait) [19, 24].

Another app, with specific focus on ataxia, is called the SARA^home^ app. This app was reported using a tablet, but the authors specifically state that it can also be used on a smartphone. SARA^home^ measures ataxia severity using the home-based, video assessments of five selected items (gait, stance, finger-to-nose test, fast alternating hand movements, speech) from the SARA [25]. These videos are reviewed by a trained rater based on the SARA scale [21].

Several publications have already implemented the use of smartphones with a range of varied methodologies that were not app based. For instance, mVEGAS [26] is a system that combines body-fixed feet inertial sensors with a smartphone-based video and a stable spatial calibration frame (two ground-fixed calibration lines). The video capture is mounted on the chest for assessment of spatiotemporal parameters of gait sequences. The sensors measure pitch angular velocity of the forefoot. Jabri and colleagues have [16] focussed on vibrotactile training utilising the internal smartphone inertial measurement unit. They used this in the context of training posture with biofeedback and implemented an independent IMU on the back to assess balance as a performance outcome measure. Smooth pursuit eye movements have been recorded and examined using mobile phone videos [23] and video recordings of horizontal saccades via mobile phones looked at oculomotor dysmetria using innovative signal processing and machine learning [22].

We also identified several publications that investigated the use of smartphone sensors for clinical domains that had potential for application to ataxias, for example gait characterised using a wearable embedded smartphone camera with a detachable lens worn by the individual on their waist [27]. Finally, we identified several publications that did not use smartphone sensors per se but used other devices such as tablets specifically to assess ataxia. These included several studies using body worn sensors combined with a tablet rather than smartphone sensor to perform the finger nose test [28, 29] and one that investigated tremor in ataxia by assessing spiral drawing [30].

### Stage 2: Delphi Survey 1: Current Smartphone Use Across AGI WG4

**Fig. 2:**
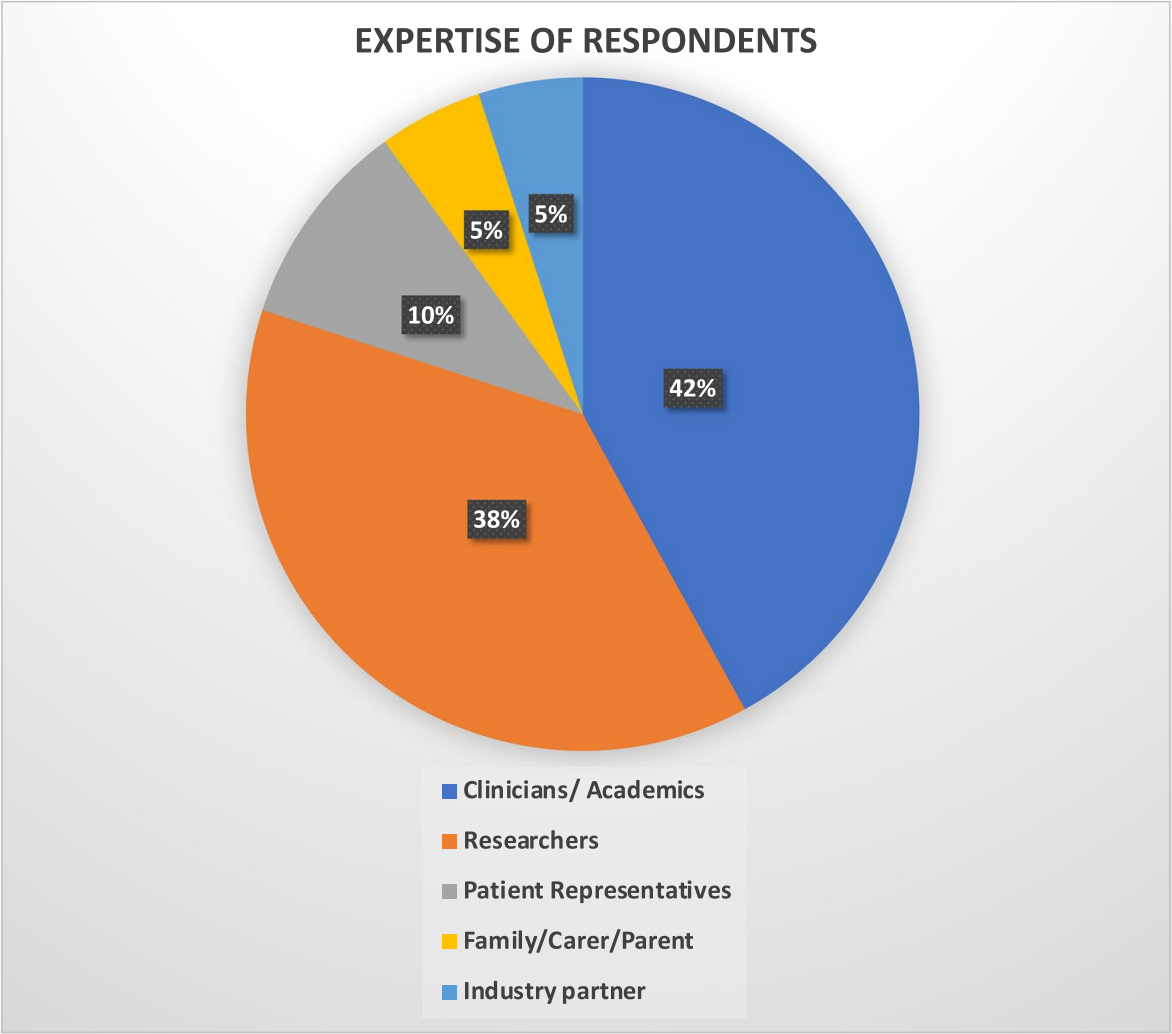
Pie chart representation of expertise, as a percentage of Delphi 1 respondents across AGI WG4 membership

We received 40/80 responses, representing 11 countries (USA/Canada/Europe/Australasia). The background expertise of respondents is shown in Fig. 2. 65% were already using a smartphone app in some healthcare capacity and iPhone apps were slightly more common than Android (53% vs 44%, other 3%).

**Fig. 3:**
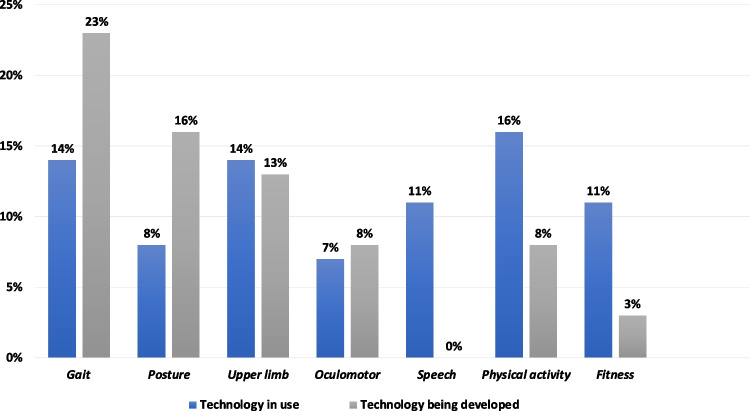
Percentages of AGI WG4 membership measuring each clinical domain with current technology vs technology in development. Blue = current technology, grey = technology in development

For questions pertaining to key motor domains, a small proportion of individuals were already using some kind of smartphone technology in some capacity (less than 20% for each domain) (see Fig. 3). Notably, for gait and posture, there were several reports of new technology being developed. Three out of nine individuals were using internal smartphone sensors; the remainder were using smartphone apps receiving data from external sensors such as smartwatches or similar.

Freetext responses included a wide variety of suggestions for outcome measures: these still mainly aligned to performance outcome measures for the Digital-Motor Working Groups (gait/balance, upper limb measures, oculomotor measures, and speech) but additional suggestions included numerous other domains such as: sleep, hearing, vision, heart rate, memory, general activity measures, and PROMs such as falls, pain, fatigue, mood, task performance (e.g. using a phone) and clinician-reported outcome measures such as well-established rating scales [25, 31, 32]. With respect to the patient population represented by this small Working Group, there were at least 1000 patients under their care, indicating a substantial reach for future studies and clinical trials.

### Stage 3: Delphi Survey 2: Priority Core Smartphone Sensor Measures Recommended for Key Domains

As described in the methodology section, we requested that key domain leads provide core priority measures based on the results from their own WGs. This identified seven priority measures in 3/4 key clinical domains, summarised in Table 1.

**Table 1:**
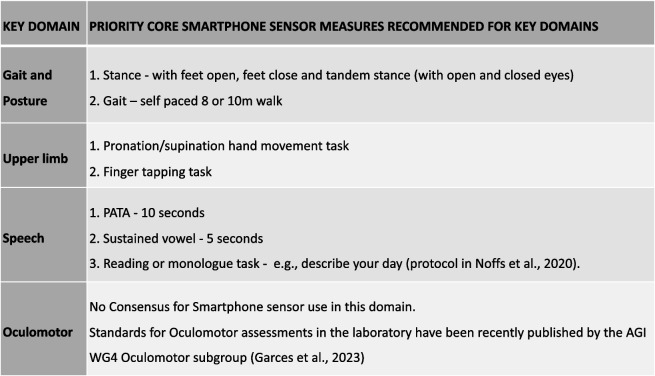
Priority smartphone sensor measures recommended by WG4 key domain leads

For gait and posture, recommendations were relatively specific and included a self-paced 8 or 10-m walk plus stance assessed with feet apart, feet close together, and tandem stance (with open and closed eyes). For upper limb, the recommendations comprised an alternating hand pronation/supination task and standardised finger-tapping task based on existing WG4 experience. In addition, the upper limb working group also highlighted that a standardised, functionally relevant multi-joint motor task would present a most promising future development, but will need validation. For speech, there were also specific priorities including the “PATA” test for 10 s, sustained vowel test for 5 s, and a reading or monologue task such as 'describe your day'. The protocol for these speech tests are in [33].

No priority measures were identified for the oculomotor domain, due to insufficient evidence for current smartphone sensor capabilities in this domain, although overall recommendations for oculomotor assessment have recently been published [9, 34].

### Stage 4: Recommendations for Standard Criteria to be Used During Smartphone Sensor Measures Based on COSMIN and COMET Principles and Regulatory Guidelines

Using the data from Stages 1–3 as well as including international standards and digital regulatory guidelines (see URLs), the Smartphone Working Group gained consensus on the critical standard criteria to be used during ataxia assessments to ensure that high quality smartphone sensor data is generated for ataxia clinical trials. We included a working definition for each criterion with input from PPIE to enable inclusion of PPIE in the process (see Table 2).

**Table 2:**
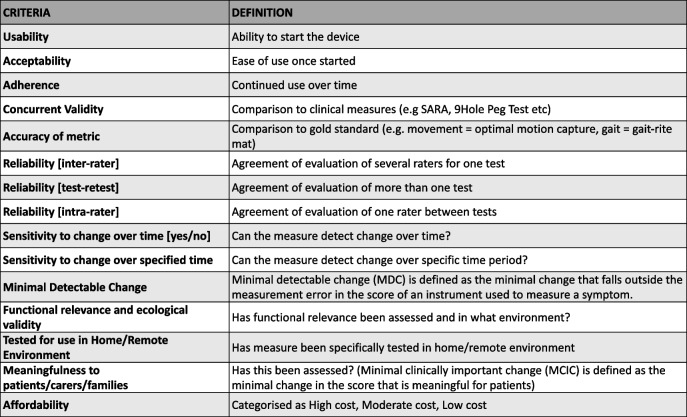
Consensus standard criteria to be used during smartphone ataxia assessments

In addition to the standard criteria, there was consensus that smartphone studies should adhere to the following principles: secure data privacy transfer that is compliant with GDPR/DPA or similar regulatory frameworks [14]. Additional areas that were outside the remit of the current study included IT interoperability and data integration, technical support, and updates.

### Stage 5: Evaluating How Many Publications to Date Using Smartphone Sensor Measures for Ataxia, Meet the Standard Criteria Developed for this Consensus paper

We investigated the extent to which the standard criteria we developed had been met in the 7 publications identified in Stage 1. On mapping the data available from each publication, we found that there was an emphasis on concurrent validity, but few other standard criteria were being routinely addressed (see Table 3). Several publications considered the importance of issues of usability by patients, but none formally studied this.

**Table 3:**
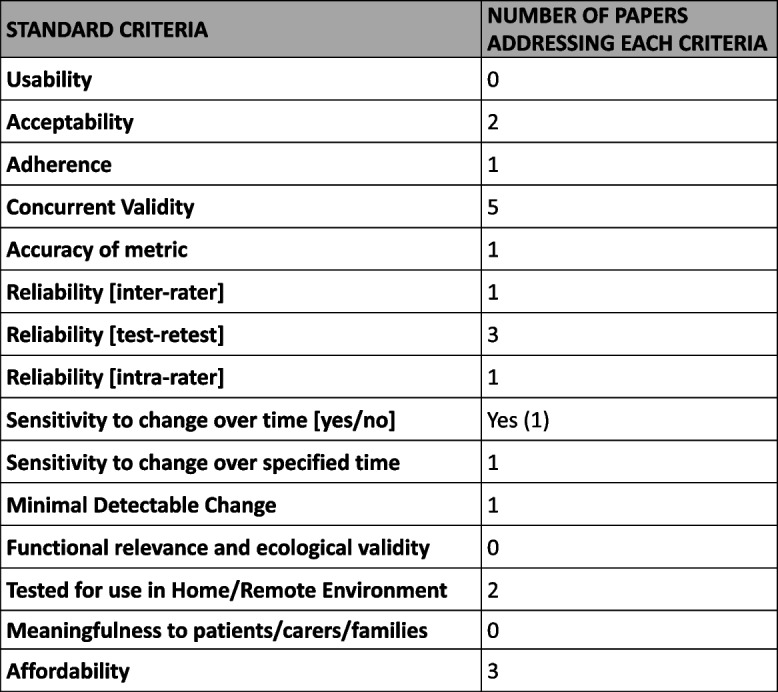
Number of papers identified in Stage 1 which address standard criteria

## Discussion and Conclusions

The ataxia field is gathering momentum. The successful identification of numerous gene mutations causing ataxia provides the promise of rational treatment avenues based on the knowledge of molecular mechanisms. The recent approval of Omaveloxolone for Friedreich’s ataxia and various RNA-targeting trials marks a new era of clinical trials for these conditions [35]. In addition, there is much interest in more wide-ranging aspects of health maintenance using movement-based interventions such as physiotherapy or exercise and the integration of pharma and non-pharma therapies.

Outcome measures play a critical role in determining the success of clinical trials in any setting, yet identifying the most appropriate set of measures in a highly heterogeneous group of disorders is extremely challenging. Digital health technologies, including those for performance outcome measures, offer a novel set of objective methodologies that have the potential to be affordable, valid and accurate, reliable, easy to use, and enable long term adherence of more frequent measurement by patients.

It is therefore timely to consider the current state of smartphone sensor use and where it is heading to provide guidance for their use that can be widely used by research teams.

Our literature review, in Stage 1, identified only a small number of papers that specifically used internal smartphone sensors to study ataxia, and several more that used another type of portable digital system that could eventually use a smartphone. These innovative publications were all from the last 3 years, demonstrating the emerging nature of this field. It was notable that there was such a wide range of devices and technologies in use, even in this small study area, and in our view, it indicates the urgent need for harmonisation and consideration of interoperability, if multi-centre, multi-national trials for patients with rare ataxias are to be successful in the future. The small number of publications may also reflect current funding priorities in other conditions such as Parkinson’s disease and multiple sclerosis. Ataxias will need significant funding to match the expertise obtained in these other areas.

As might be expected, the majority of smartphone papers investigated gait and balance (4/7). Only one paper specifically investigated the upper limb movements, and the two papers investigating the eye movements are very recent. With respect to speech evaluation, microphones within smartphones are undergoing constant innovation, as are touch screens, cameras, and IMU sensors, and this should be acknowledged alongside recognition that this is a rapidly changing speciality, with numerous factors required to optimise signal quality, mitigate environmental noise, and devise protocols that can capture wide variations in severity of disease [33]. Currently, we identified only one study analysing speech with a smartphone (as part of the SARA^home^ app) and further developments and protocols will be required to fully utilise smartphone sensors for speech assessments.

The Delphi 1 survey in Stage 2 revealed a huge network of experts who are devoting their time and expertise to the field of ataxias. The survey revealed wide variations in use of digital health technologies, mirroring the studies identified in the literature review and again highlighting the importance of harmonisation, particularly because individual genetic subtypes of ataxias can be very rare. In addition, the Delphi 1 highlighted that researchers were ultimately aiming to utilise all smartphone sensors, including microphone, camera/video, touchscreen, and IMUs.

In Stage 3, the Delphi 2 survey not only revealed the extent to which some of the key domain groups have made progress in identifying key priorities for ataxia assessments but also revealed the limited data set available with which to evaluate the use of smartphone sensors. There was no consensus for oculomotor assessments, due to current limitations of the technologies available. Further research is particularly required in this domain. Recently, two papers have published a core set of quantitative oculomotor paradigms and parameters for clinical studies of (hereditary) ataxias. [9, 34]. Importantly, the use of commercially available, mobile recording devices with recording frequencies above 100 Hz and based on video-oculography is strongly recommended for oculomotor assessments, but further work is needed for smartphone use.

Using an iterative and interactive process involving the Expert panel, in Stage 4, we were able to define 15 standard criteria that outcome measures from internal smartphone sensors should meet. The aim of defining these criteria is to provide a platform of basic standards for measurement that will give confidence in the valid use of smartphone sensors in clinical trials and to enable harmonisation across trials to support meta-analysis and synthesis of research findings. We also highlight the critical need, when implementing measures, for utilising clear standardised protocols in order to obtain accurate, valid, and reliable data from measures, whether they are used in clinical or community settings.

In Stage 5, we assessed the small literature identified in Stage 1 and mapped the types of assessments performed in each study against our newly devised standard criteria. The most common criteria assessed by the publications were validity. This illustrates just how early in development the technology of smartphone sensors is for use in ataxias and underscores the necessity of further studies which adhere to the standard criteria so that smartphone sensors can be confidently used in clinical trials. We noted that there were few studies measuring the functional relevance of measures to patients, despite meaningfulness to patients being widely recommended by Regulatory agencies. Furthermore, there were few longitudinal studies, a notable omission given that clinical trials of degenerative neurological diseases need longitudinal data to assess efficacy, and likely reflecting the focus on validation, and possibly also indicating limited funding support. Clearly, there is an urgency to validate metrics for sensitivity to change, requiring a co-ordinated approach and appropriate funding to support such studies.

In addition to the published literature, we also considered apps in current use in the ataxia field. A key example of a smartphone App specifically developed for one specific type of ataxia is “The FA App” (see https://www.thefaapp.org), aimed at furthering research into Friedreich Ataxia. The FA App is currently available for both iOS and Android and is in use by 2748 users out of which 1369 have FA. These users are spread globally over 98 countries and are being serviced in 9 different languages. Apart from various community features and a news section, “The FA App” also rolled out so-called virtual trials in which both people with FA as well as controls participated. The App tracked them in a mood survey (QoL), a tapping game, as well as their speech. The initial results of “The FA App” were presented at the ICAR 2022 congress in Addison, TX, USA. However, at the time of writing, this data has not yet been published.

## Strengths and Limitations

This study represents a first effort towards establishing consensus guidance for utilising smartphone sensors in ataxia clinical trials. We have conducted a literature review and identified several papers reporting important and innovative methodologies for measuring ataxia motor features. We noted that reported work of a highly varied nature has focussed on validation which is clearly essential. However, to make progress in international collaborations for clinical trials, methodologies will need standardisation. We surveyed the membership of AGI WG4 to establish expertise among the members and determined their use of smartphone sensors and related technologies, identified 7 priority areas for ataxia digital motor assessments and developed 15 standard criteria for use in future research. These findings form the basis of our guidance. We have represented clinicians, researchers, patient representatives, and Industry across a very wide geographical area, including 11 countries within WG4 and 3 additional countries represented by external expert reviewers.

However, it is important to acknowledge several limitations to this work. First, the surveys represent those members of the AGI WG4 who responded, and we do not have data on non-responders. Second, we could only identify 7 publications that investigated motor features of ataxia using internal smartphone sensors and different types of sensors (accelerometer/gyroscope, in-built camera/digital video, and microphone) were employed in each study, making cross comparison difficult. Third, our analysis was limited to English language studies, potentially excluding valuable insights from non-English publications. In future smartphone sensor studies, a focus on usability and adherence, relevance to patients and longitudinal studies, is vital. Moreover, ethical implications, including data privacy and security, should be thoroughly evaluated when using smartphone sensors in clinical trials.

## Conclusions

In conclusion, we have developed consensus guidance of priority measures in ataxia using smartphones and standard criteria for measurement properties that should be addressed in all future smartphone sensors research studies. This consensus will enable comparison of studies across centres and internationally, ensuring harmonisation for future clinical trials and more efficient use of research data that can be reliably synthesised in metanalysis.

## Data Availability

Not applicable.

## Abbreviations

AGI: Ataxia Global Initiative
WG: Working Groups
WG4: Digital-Motor Biomarkers Working Group with 5 sub-Working Groups: 4 for pre-defined key clinical domains speech, e.g. oculomotor, upper limb, and gait/posture function, and the fifth which is the Smartphone group
PPIE: Patient and Public Involvement and Engagement
